# Retinal venous pressure: the role of endothelin

**DOI:** 10.1186/s13167-015-0043-1

**Published:** 2015-10-26

**Authors:** Josef Flammer, Katarzyna Konieczka

**Affiliations:** Department of Ophthalmology, University of Basel, Mittlere Strasse 91, CH-4031 Basel, Switzerland

**Keywords:** Retinal venous pressure (RVP), Dilated retinal veins, Endothelin-1 (ET-1), Ophthalmodynamometry, Venous constriction, Retinal vein occlusion (RVO), Diabetes mellitus, Glaucoma, Primary vascular dysregulation (PVD), Flammer syndrome (FS), Predictive, preventive and personalized medicine

## Abstract

The retinal venous pressure (RVP) can be measured non-invasively. While RVP is equal to or slightly above intraocular pressure (IOP) in healthy people, it is often markedly increased in patients with eye or systemic diseases. Beside a mechanical obstruction, the main cause of such an elevation is a local dysregulation of a retinal vein, particularly a constriction induced by endothelin-1 (ET-1). A local increase of ET-1 can result from a high plasma level, as ET-1 can diffuse from the fenestrated capillaries of the choroid into the optic nerve head (ONH), bypassing the blood retinal barrier. A local increase can also result from increased local production either by a sick neighboring artery or retinal tissue. Generally, the main factors increasing ET-1 are inflammations and hypoxia, either locally or in a remote organ. RVP is known to be increased in patients with glaucoma, retinal vein occlusion (RVO), diabetic retinopathy, high mountain disease, and primary vascular dysregulation (PVD). PVD is the major vascular component of Flammer syndrome (FS). An increase of RVP decreases perfusion pressure, which heightens the risk for hypoxia. An increase of RVP also elevates transmural pressure, which in turn heightens the risk for retinal edema. In patients with RVO, a high level of RVP may not only be a consequence but also a potential cause of the occlusion; therefore, it risks causing a vicious circle. Narrow retinal arteries and particularly dilated retinal veins are known risk indicators for future cardiovascular events. As the major cause for such a retinal venous dilatation is an increased RVP, RVP may likely turn out to be an even stronger predictor.

## Review

### Introduction

A few years ago, we described the local dysregulation of retinal veins as a major cause of retinal vein occlusion (RVO) [1]. Since providing this description, several reports on retinal venous pressure (RVP) were published, motivating us to update the present knowledge about RVP in terms of its regulation and role in different diseases.

### Definition and measurement of retinal venous pressure

**Fig. 1:**
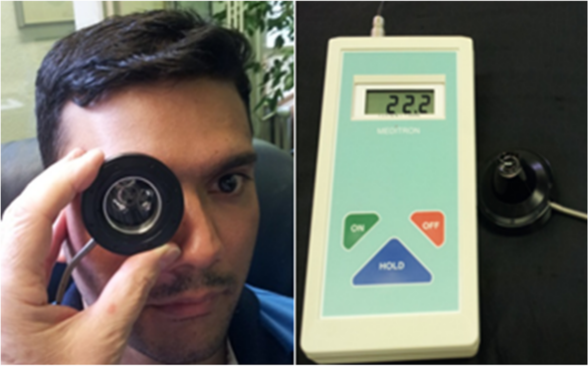
Measurement of retinal venous pressure by an ophthalmodynamometer. (Use of the device demonstrated on a co-worker of our team, with written confirmed consent for publication.)

Alongside the retinal veins, there is a gradient of the intravascular pressure of about 0.9 mmHg/mm. The pressure is higher in the periphery than in the optic nerve head (ONH) area where the vein exits the eye [2]. To measure the exact pressure in a vein, one needs to insert a cannula intraluminally—a procedure not feasible under clinical conditions. To estimate the approximate pressure in the veins, intraocular pressure (IOP) is increased until the vein pulsates. In this context, we define RVP equal to the IOP where the vein at or close to the ONH starts to pulsate. Pressure inducing a slight pulsation and pressure leading to (transient) collapse of the vein occur within a certain range. Such methodological discussion, although very important, is not a topic of this review. In most available studies, the RVP was defined as equal to the IOP inducing a slight pulsation. Why the vein pulsates is still controversial [3]. A recent analysis attributes it to the difference between IOP and cerebrospinal fluid (CSF) pressure pulsations (Levine DH, Bebie H. Phase and amplitude of spontaneous retinal vein pulsation: an extended constant input - variable output model, submitted). Most studies used the Kontaktglas-Dynamometer (also called ophthalmodynamometer) by Dr. Loew [4] (IMEDOS Systems UG, Jena, Germany) (Fig. 1).

### Normal RVP values

The RVP generally is not (or is only for a short period of time) lower than the IOP. If it were, the veins would collapse. However, based on the Starling law, the transmural venous pressure is probably temporarily negative at the exit from the eye [2].

**Fig. 2:**
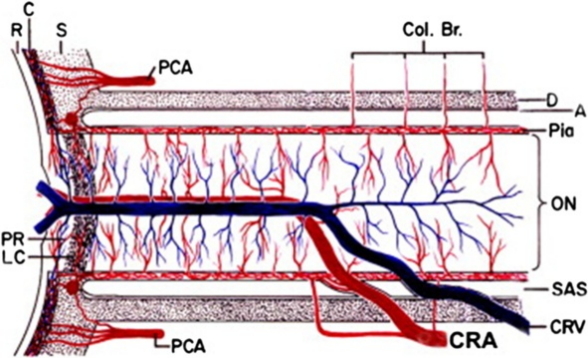
Schematic representation of the blood vessels in the optic nerve (from [49], with permission). The central retinal vein runs through the SAS; therefore, retinal venous pressure is equal or higher than the pressure in the SAS. Abbreviations: *A* arachnoid, *C* choroid, *CRA* central retinal artery, *Col. Br.* collateral branches, *CRV* central retinal vein, *D* dura, *LC* lamina cribrosa, *ON* optic nerve, *PCA* posterior ciliary artery, *PR* prelaminar region, *R* retina, *S* sclera, *SAS* subarachnoid space

The central retinal vein leaves the eye through the anterior part of the optic nerve (ON) (Fig. 2), runs through the CSF, and penetrates the meninges of the ON. Therefore, RVP can also not be lower than the CSF pressure around the ON [5].

In healthy subjects, a spontaneous retinal venous pulsation can often be observed, indicating that the RVP in these subjects corresponds more or less to spontaneous IOP. The ratio of healthy subjects with spontaneous retinal venous pulsation varies from 75 % [6] to 98 % [7]. It is important to note that RVP can be above IOP even in presumed healthy subjects.

### Mechanism leading to high RVP

**Fig. 3:**
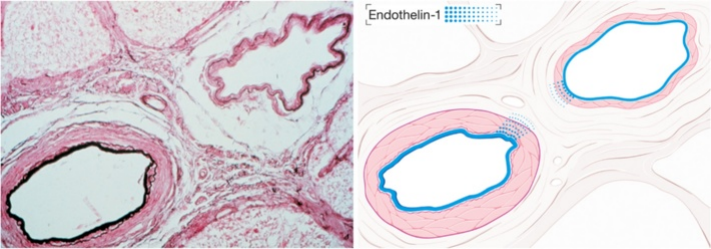
Arteriovenous crossing in a human retina (reproduced from [1]). Retinal arteries and veins are very close both in the optic nerve head and at the arteriovenous crossing in the retina. This allows molecular cross talk between arteries and veins in these specific locations. *Left*: microscopic view of a histologic specimen. *Right*: schematic representation of the feasibility of a molecular interference. In physiological conditions, the locally produced endothelin-1 has an effect just on the underlying smooth muscle cells. This is different under pathological conditions (see also Fig. 8)

There are a number of principal mechanisms leading to a high RVP:

1. *IOP increase*: High IOP automatically leads to a high RVP. This is particularly relevant in situations with a marked elevated IOP, such as in patients with acute angle closure glaucoma.
2. *Increased CSF pressure*: This is relevant in subjects with CSF pressure higher than IOP. A number of authors even use RVP to estimate CSF pressure [8].
3. *Retinal venous thrombosis*: If the outflow is blocked, e.g., by a thrombus, the increased resistance to flow increases RVP [9]. A real thrombus, however, is—in contrast to earlier assumptions—a rare event, and even in its event, it may rather be secondary to blood flow deceleration [1].
4. *Compression of the retinal vein by a distorted lamina cribrosa*: Such a compression could partly explain the relationship between RVP and the stage of glaucoma [10]. However, we agree with Hayreh [11] that this mechanism may be an exception.
5. *Compression of a vein by a bypassing artery*: At the crossings in the retina and at the level of the lamina cribrosa, arteries and veins come close to and even share a common adventitia. It has therefore been assumed that veins can be crushed mechanically by sclerotic arteries and that this may even lead to a vein occlusion [12]. We have postulated that this narrowing of a vein may rather be due to active constriction than due to a mechanical compression, inter alia, mediated by molecular cross talk between arteries and veins [1] (Fig. 3). Our hypothesis is supported by findings with optical coherence tomography (OCT) (Fig. 4) [13] and with adaptive optics [14].

**Fig. 4:**
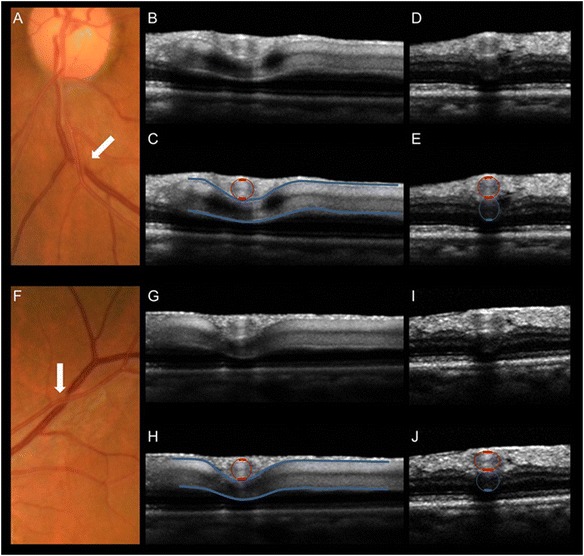
Venous narrowing rather than compression at the arteriovenous crossings. **a, f** Fundus photographs of an arteriovenous (AV) crossing, showing crossing phenomena (*arrows*). **b, g** Optical coherence tomography (OCT) sections obtained along the retinal veins at crossings. **d, i** OCT sections obtained at the crossing, perpendicular to the retinal veins. **c, e, h, j** OCT images with indications of vascular outlines from the upper OCT images. **a–e** An AV crossing that shows concealment. The retinal vein shows focal narrowing of the lumen at the crossing. However, the vein does not exhibit signs of compression or flattening. The venous lumen is round, even just under the artery. **f–j** An AV crossing that shows severe tapering. On fundus photographs, the bloodstream seems to be extremely narrow in the area of the crossing site. OCT sections reveal that the actual venous lumen is larger than portrayed by fundus photograph and maintains a round shape. *Red lines* indicate arterial outlines, and *blue lines* indicate venous outlines. (Reproduced from [13], with permission)
6. *Increased level of endothelin-1* (*ET*-*1*): The retinal vessels lack autonomic innervation. The main regulators of the size of retinal vessels are the vascular endothelial cells (VEC). VEC dysfunction occurs in many conditions, e.g., in glaucoma [15]. However, it is not likely that a general endotheliopathy leads to such a localized constriction. Circulating vasoactive hormones have limited impact on the size of retinal vessels as long as the blood-retina barrier (BRB) is intact. Vasoactive substances have, however, a major impact if they reach retinal vessels from the outside, which gives them direct contact with the smooth muscle cells and pericytes. The likelihood that vasoactive molecules, such as ET-1, reach the vessels from the outside is particularly high in and around the ONH. Together with the fact that veins respond to a lower dose of ET-1 than arteries [16], it is evident that retinal veins in the area of the ONH are relatively often constricted.

### Conditions leading to an ET-1 increase

**Fig. 5:**
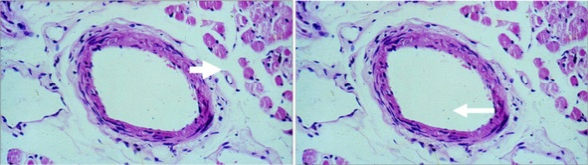
Release of endothelin-1 (ET-1). The vascular endothelial cells release the major part of ET-1 abluminally (for local action) and a smaller part intraluminally, influencing the concentration in the circulating blood (from [50], with permission)

ET-1 is a hormone which acts primarily locally. The VEC release the major part of ET-1 abluminally for the local smooth muscle cells and just a smaller part intraluminally, influencing the concentration in the circulating blood (Fig. 5).

While VEC produce ET-1 under a physiological condition, basically all cells produce ET-1 if they are under stress. There are two main causes:

1. *Hypoxia*: Hypoxia leads to an increase of hypoxia-inducible factor-1 alpha (HIF-1-alpha), a transcription factor. This leads to the upregulation of several genes, such as erythropoietin, vascular endothelial growth factor (VEGF), and ET-1 [17] (Fig. 6). Hypoxia somewhere in the body can increase ET-1 in the circulating blood and thereby induce a secondary vascular dysregulation in the eye [18]. A local hypoxia in the eye also increases ET-1 in the eye. This explains why a bilateral internal carotid artery stenosis can induce a central retinal vein occlusion (CRVO) [19].

**Fig. 6:**
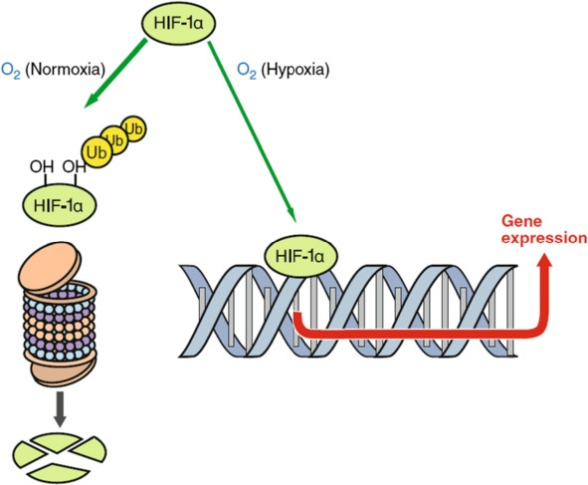
Hypoxia-inducible factor-1 alpha (HIF-1-alpha). If oxygen concentration in a cell is lowered, less HIF-1 alpha is oxidized and degraded. Thus, more HIF-1 alpha can move into the nucleus, where it acts as a transcription factor for vascular endothelial growth factor, endothelin-1, and others (from [50], with permission)
2. *Inflammations*: Most types of inflammations, particularly autoimmune-mediated inflammations (such as giant cell arteritis [20]), lead to an increased production of ET-1 and thereby induce secondary vascular dysregulation in remote organs, particularly in the eye. In addition, endothelin (ET) receptor blockers have proven to have an anti-inflammatory effect in uveitis [21].

### The functional local constriction of the retinal veins

**Fig. 7:**
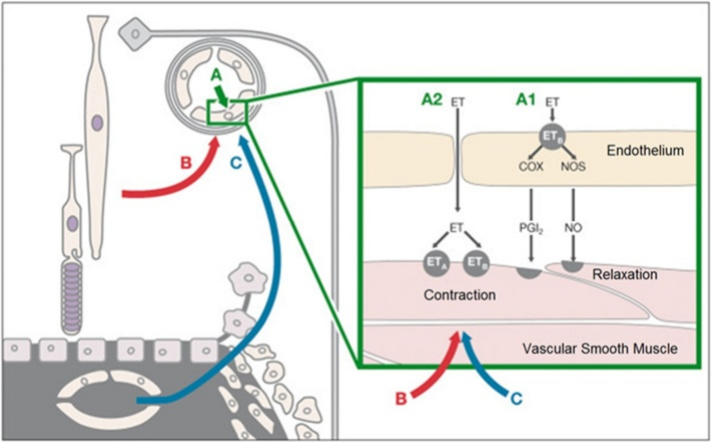
Influence of endothelin-1 (ET-1) on blood vessels in the optic nerve head and adjacent retina (reproduced from [31]). (*A*) Circulating ET-1 reaches just the endothelial cells (*A1*) and has a more or less neutral effect on the size of the vessels. If the blood-brain barrier is disrupted (*A2*), it reaches the vascular smooth muscle cells directly and induces vasoconstriction. (*B*) Hypoxic retina produces ET-1, which diffuses also to neighboring vessels. (*C*) ET-1 diffuses from fenestrated capillaries of the choroid into the optic nerve head and adjacent retina. This can induce a local constriction of the veins and in turn increase retinal venous pressure

A local constriction of retinal veins in the area of the ONH increases RVP. Such a local constriction can be induced by a local increase of ET-1. As already mentioned, there are three potential sources of ET reaching the retinal veins from the outside (Fig. 7).

1. *Circulating blood*: The ET in the blood can directly reach the smooth muscle cells of retinal vessels with three modalities: (a) diffusion from fenestrated capillaries of the choroid into the ONH, (b) diffusion from the microvessels of the prelaminar portion of the ONH because these vessels lack normal properties of blood-brain barrier [22], and (c) diffusion from local vessels in case of a breakdown of the BRB. Therefore, an increase of ET concentration in the circulating blood has an impact on the retinal veins in healthy eyes and an even greater impact in the presence of local pathology.
2. *Diseased arteries*: These can produce increased amounts of ET-1. One part of it is secreted abluminally, reaching veins from the outside in areas where the arteries and veins are close, i.e., in the ONH and at the arteriovenous crossings (Fig. 8 (left)).

**Fig. 8:**
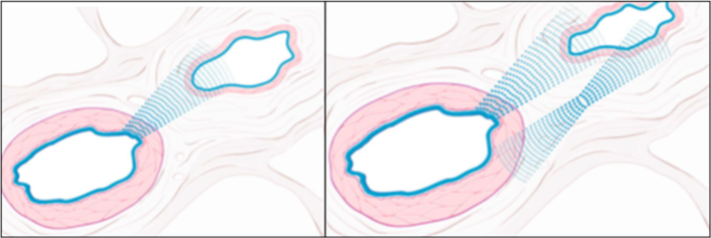
Schematic representation of an arteriovenous crossing in retina in pathological conditions (reproduced from [1]). *Left*: a diseased artery produces a higher amount of endothelin-1 (ET-1) inducing a venous constriction. *Right*: local hypoxia further contributes to the ET-1 level and to the constriction of the vein. This can eventually lead to an RVO
3. *Hypoxic neighboring tissue* (*Fig.**8* (*right*)): Hypoxia increases ET-1 in any tissue including the eye. This explains why disturbed ocular blood flow due to arterial diseases can lead to a secondary increase of RVP or even to RVO [19].

### Increased RVP in different diseases

Available studies on RVP are still limited; therefore, the behavior of RVP in most diseases is still unknown. In the following sections, we summarize existing information.

### Retinal vein occlusion

**Fig. 9:**
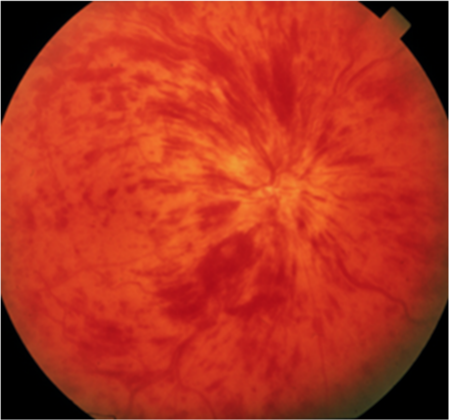
Clinical picture of retinal vein occlusion

RVO (Fig. 9) occur in older subjects with risk factors, such as arterial hypertension or increased IOP, and in younger subjects, particularly those suffering from Flammer syndrome (FS) [23].

**Fig. 10:**
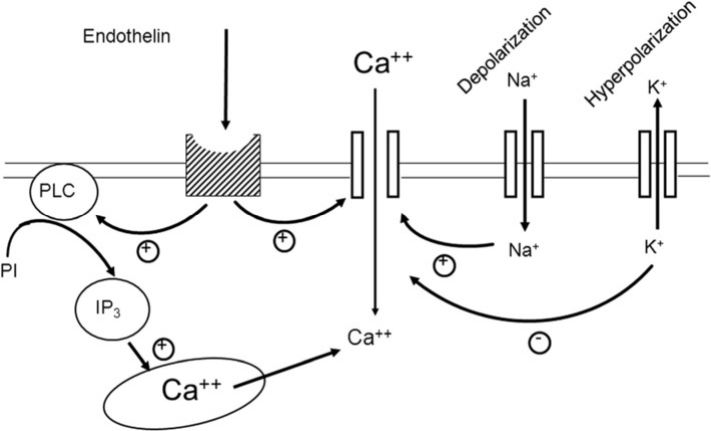
Stimulation of endothelin receptors. Endothelin stimulates the G-coupled endothelin receptors A. This leads to both opening of the calcium channels and liberation of calcium from internal stores (from [51], with permission)

RVP is increased in eyes with RVO [24], more in the ischemic than the non-ischemic type [25]. Surprisingly, however, RVP is also often increased in the clinically non-affected fellow eye of such patients [26]. This can be explained by the increased plasma ET-1 of such patients [27, 28]. The role of ET-1 is further supported by the observation that calcium channel blockers (CCB) decrease RVP [29]. The ET receptor type A is a G-protein-coupled receptor. Its stimulation leads to both an influx of calcium from the outside of the cell and to a liberation of calcium from the internal storages. The CCB inhibit the first component (Fig. 10) and thereby reduce, but do not totally eliminate, the vasoconstrictive effect of ET-1.

**Fig. 11:**
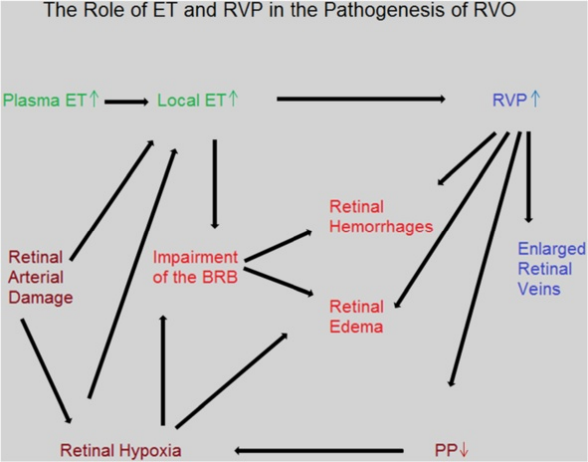
The role of endothelin and retinal venous pressure in the pathogenesis of retinal vein occlusion (RVO). The scheme indicates the influence of ET-1 on RVP. Increased RVP reduces PP. If this reduction is not compensated enough by arterial vasodilation, hypoxia will result, inducing possibly a vicious circle that potentially ends in the clinical picture of an RVO. Abbreviations: *ET-1* endothelin-1, *RVP* retinal venous pressure, *PP* perfusion pressure, *BRB* blood-retina barrier

We assume that RVP slowly increases in patients developing RVO (as a consequence of a local vein constriction) and thereby progressively decreases perfusion pressure (PP). If PP drops below a critical limit, the hypoxic retina starts to further increase the local ET-1 level, with the danger for a vicious circle that includes a further decrease of PP and a breakdown of the BRB [30]. This in turn leads to retinal hemorrhages [31] and retinal edema. The hypothetical relationship between ET-1, RVP, and RVO is depicted in Fig. 11.

### Diabetes mellitus

**Fig. 12:**
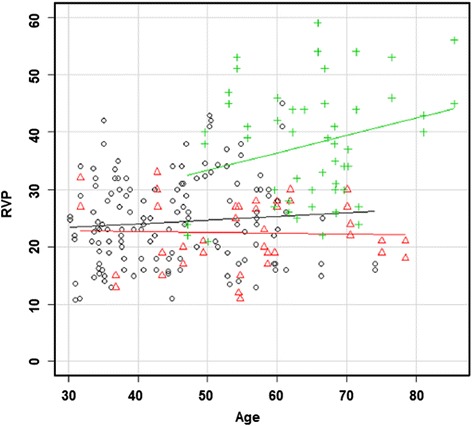
Retinal venous pressure (RVP) in patients with and without diabetic retinopathy (DR). RVP is increased in patients with DR but not in diabetes patients without DR. RVP (mmHg) is plotted with the age (years). *Straight lines* indicate regression slopes. The *black circle* indicates the control group, the *red triangle* represents the group diabetes patients without DR, and the *green cross* represents patients with DR (reproduced from [35])

Patients with diabetes mellitus (DM) often have an increased level of ET-1 [32], retinal vessel tortuosity [33], and dilated retinal veins [34]. Recently, it has been reported that DM patients with diabetic retinopathy (DR) have higher RVP than DM patients without DR and healthy controls (Fig. 12) [35]. Although the causal relationship is not yet clear, ET-1 most probably plays a crucial role. This increased RVP reduces PP and therefore increases the risk for hypoxia contributing to a vicious circle. Furthermore, high RVP increases transmural pressure and therefore amplifies the risk for retinal edema.

### Glaucoma

A number of studies have shown that a reduced PP and increased fluctuation of PP are risk factors for glaucoma progression [36]. For the calculation of PP, RVP was assumed to be equal to IOP in most studies. Meanwhile, it has been shown that in the majority of glaucoma patients, RVP is far above IOP [37]; therefore, the PP in such cases is much lower than was previously assumed [10].

**Fig. 13:**
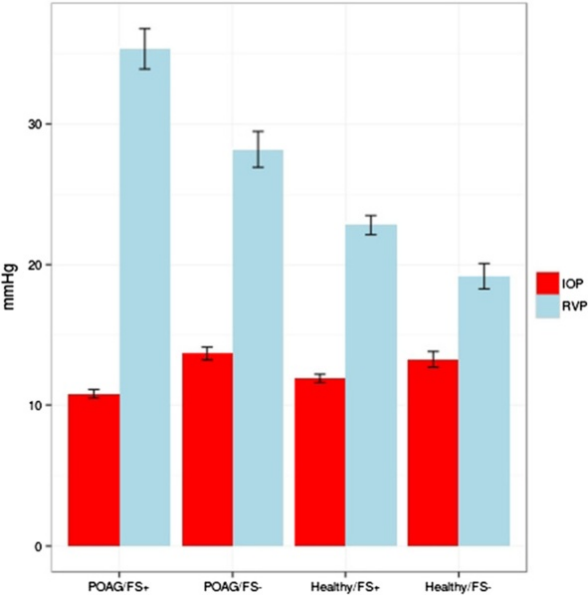
The influence of Flammer syndrome (FS) on retinal venous pressure (RVP). Healthy subjects with FS have slightly higher RVP than healthy subjects without FS. In glaucoma patients, this difference was even larger; the difference between IOP and RVP was greatest in subjects suffering from both glaucoma and FS. Abbreviations: *IOP* intraocular pressure, *RVP* retinal venous pressure, *POAG/FS+* patients with glaucoma and FS, *POAG/FS-* patients with glaucoma and without FS, *Healthy/FS+* healthy subjects with FS, *Healthy/FS-* healthy subjects without FS (reproduced from [38])

Healthy subjects with FS have slightly higher RVP than healthy subjects without FS. In glaucoma patients, this difference is even larger. The difference between IOP and RVP was greatest in subjects suffering from both glaucoma and FS [38] (Fig. 13).

Interestingly, the RVP in glaucomatous eyes with splinter hemorrhages is not increased [39]. This supports our hypothesis that these hemorrhages are not due to ruptures of vessels but rather a consequence of a locally disturbed BRB [31]. Indeed, the difference between brachial arterial blood pressure and the retinal arterial blood pressure was higher in the eyes with hemorrhages, indicating a higher blood flow resistivity in the arteries somewhere between the heart and the eye, most probably in the retroocular arteries [40]. This, in turn, increases the probability for overall hypoxia in the eye and thereby a general increase of ET-1 in the eye. This leads to a dysfunction of the BRB [31] but not to a very local constriction of the retinal veins and therefore not necessarily to RVP increase.

### High-altitude retinopathy

It has long been known that high altitude leads in some subjects to a swollen ONH, dilated retinal veins, and retinal hemorrhages [41]. These changes have been assumed to be secondary to an increase of intracranial pressure due to cerebral edema [42]. However, high altitudes lead to a mild but global hypoxia and therefore to an increased ET-1 level in the circulating blood [17, 43] and an RVP increase [4]. Subjects with primary vascular dysregulation (PVD), the vascular component of a FS, seem to react more often and more intensively to high altitudes [44]. We observed that a young lady with FS repeatedly developed mild retinal venous stasis syndrome after ballooning. One day she tried to cross the Alps in a balloon and lost consciousness before reaching the required height.

### Occlusion of a cilioretinal artery

**Fig. 14:**
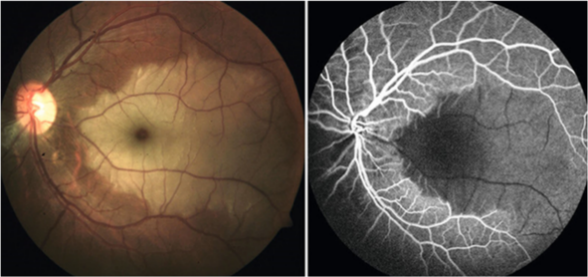
Occlusion of a cilioretinal artery (CLRAO) (from [45], with permission). *Left*: fundus with white ischemic retina with a “cherry-red spot” appearance at the macula in an area supplied by a cilioretinal artery. *Right*: corresponding fluorescein angiogram

An RVP increase has an impact on ONH perfusion (as this area is drained by the central retinal vein) and is therefore relevant for glaucoma. However, it influences also a portion of the choroidal/retinal circulation in cases with cilioretinal arteries. Indeed, an occlusion of a cilioretinal artery (CLRAO) has been reported in a patient with increased RVP and FS [45] (Fig. 14), and central retinal vein occlusions are sometimes associated with CLRAO.

### Inflammatory diseases

**Fig. 15:**
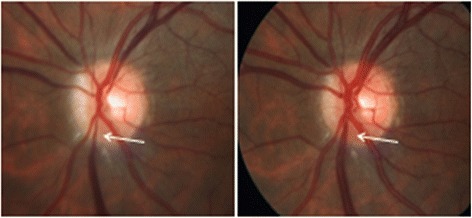
Changes of the diameter of the retinal veins over time. The temporal dynamic of the size of the veins is illustrated in a patient with Takayasu arteritis. *Left*: during acute inflammation (of the aorta), the optic nerve head (ONH) of this young patient was pale, and the retinal veins at the border of the ONH were markedly constricted. *Right*: after treatment of the inflammation, the ONH colored again, and the veins reached nearly normal sizes. *Arrows* indicate one of these veins

Autoimmune diseases, such as giant cell arteritis, can lead to both increased ET-1 levels and the upregulation of ET receptors [20, 46]. Interestingly, in patients with giant cell arteritis, an anterior ischemic optic neuropathy (AION) occurs relatively often, although giant cell arteritis is a disease of the medium-sized and large arteries and not of the arterioles. We have hypothesized that the AION in such patients may not, or at least not always, be the consequence of locally inflamed vessels but rather the consequence of a secondary dysregulation of these vessels, which is induced by ET-1 produced by remote inflamed vessels [18, 31]. We recently had the chance to observe a patient with Takayasu arteritis, a vasculitis primarily affecting the aorta but also leading to increased ET-1 plasma levels [47] and sometimes even to a pulmonary hypertension [48]. During phases of acute inflammation, the ONH of our young patient turned pale and the retinal veins in the area of the ONH markedly constricted. After treatments of the inflammation with an anti-IL6 agent, the vision improved, ONH colored again, and the arteries but particularly the veins reached nearly normal sizes again (Fig. 15).

## Conclusions

Clinically, RVP can be measured at least approximatively. Despite a number of limitations of a currently available methodology to measure RVP, the ophthalmodynamometry produces helpful results. Based on the studies summarized in this review, we can conclude that, beside mechanical obstruction, the main cause of an RVP increase is a local vein constriction, mainly mediated by ET-1. In the long run, ET-1 not only constricts vessels but also induces tissue remodeling of the vessel wall. Therefore, the impediment to flow may then have functional and structural components. ET-1 can diffuse from the circulating blood into the ONH via the choroid. ET-1, however, can also diffuse from a diseased artery into the wall of a neighboring vein. Finally, ET-1 is also produced locally from hypoxic tissue. In most healthy subjects, RVP is close to IOP or slightly above. RVP is, however, distinctly increased in some ocular and systemic diseases, particularly diseases accompanied by inflammation or hypoxia. This is of major clinical relevance for the following reasons:

1. High RVP reduces the PP and therefore reduces circulation of both the retina and the ONH. The reduction of retinal circulation is relevant for diseases, such as RVO or DR. The reduction of ONH perfusion contributes to glaucomatous damage.
2. Augmented RVP increases transmural pressure and therefore elevates the risk for retinal edema. Unfortunately, very little has been known until now about the relationship between retinal edema and the RVP and whether the pharmacological reduction of RVP reduces the edema.
3. RVP might be a very sensitive marker for systemic conditions associated with elevated ET-1. If this is confirmed in future studies, RVP will be a sensitive clinical marker for corresponding diseases and may also be helpful for follow up.
4. Patients with systemic hypertension have retinal veins relatively wider to retinal arteries, and generally narrow retinal arteries and particularly dilated retinal veins are proven risk indicators for future cardiovascular diseases [34]. The reason why dilated veins are such a strong risk indicator was unexplained until now. We assume that the dilatation of the retinal veins is at least partly a consequence of a high RVP. Therefore, a high RVP might turn out to be an even stronger predictor for future cardiovascular events.

At present, we are still in the early stage of research in this field. We suggest the following steps in order to promote this research area:

- The methodology to measure RVP should and can be improved.
- The relationship between RVP and ET-1 needs to be established in experimental studies.
- Modalities (drugs) to reduce RVP should be further evaluated, first in animals and then in humans.
- Whether the reduction of RVP improves prognosis of diseases like glaucoma needs to be proven by interventional studies.
- The prognostic power of an increased RVP should be evaluated by epidemiological studies.

## Abbreviations

AION: anterior ischemic optic neuropathy
BRB: blood-retina barrier
CCB: calcium channel blockers
CLRAO: occlusion of a cilioretinal artery
CSF: cerebrospinal fluid
DM: diabetes mellitus
DR: diabetic retinopathy
ET: endothelin
ET-1: endothelin-1
FS: Flammer syndrome
HIF-1-alpha: hypoxia-inducible factor-1 alpha
IOP: intraocular pressure
OCT: optical coherence tomography
ON: optic nerve
ONH: optic nerve head
PP: perfusion pressure
PVD: primary vascular dysregulation
RVO: retinal vein occlusion
RVP: retinal venous pressure
VEC: vascular endothelial cells
VEGF: vascular endothelial growth factor
